# 3D Printing of Inertial Microfluidic Devices

**DOI:** 10.1038/s41598-020-62569-9

**Published:** 2020-04-03

**Authors:** Sajad Razavi Bazaz, Omid Rouhi, Mohammad Amin Raoufi, Fatemeh Ejeian, Mohsen Asadnia, Dayong Jin, Majid Ebrahimi Warkiani

**Affiliations:** 1 School of Biomedical Engineering, University of Technology Sydney, Sydney, NSW 2007 Australia; 2 School of Engineering, Macquarie University, Sydney, NSW 2109 Australia; 3 Institute for Biomedical Materials & Devices (IBMD), Faculty of Science, University of Technology Sydney, Sydney, NSW 2007 Australia; 4 SUStech-UTS joint Research Centre for Biomedical Materials & Devices, Southern University of Science and Technology, Shenzhen, 518055 P.R. China; 5 Institute of Molecular Medicine, Sechenov University, Moscow, 119991 Russia

**Keywords:** Biomedical engineering, Lab-on-a-chip, Fluid dynamics, Particle physics, Isolation, separation and purification

## Abstract

Inertial microfluidics has been broadly investigated, resulting in the development of various applications, mainly for particle or cell separation. Lateral migrations of these particles within a microchannel strictly depend on the channel design and its cross-section. Nonetheless, the fabrication of these microchannels is a continuous challenging issue for the microfluidic community, where the most studied channel cross-sections are limited to only rectangular and more recently trapezoidal microchannels. As a result, a huge amount of potential remains intact for other geometries with cross-sections difficult to fabricate with standard microfabrication techniques. In this study, by leveraging on benefits of additive manufacturing, we have proposed a new method for the fabrication of inertial microfluidic devices. In our proposed workflow, parts are first printed via a high-resolution DLP/SLA 3D printer and then bonded to a transparent PMMA sheet using a double-coated pressure-sensitive adhesive tape. Using this method, we have fabricated and tested a plethora of existing inertial microfluidic devices, whether in a single or multiplexed manner, such as straight, spiral, serpentine, curvilinear, and contraction-expansion arrays. Our characterizations using both particles and cells revealed that the produced chips could withstand a pressure up to 150 psi with minimum interference of the tape to the total functionality of the device and viability of cells. As a showcase of the versatility of our method, we have proposed a new spiral microchannel with right-angled triangular cross-section which is technically impossible to fabricate using the standard lithography. We are of the opinion that the method proposed in this study will open the door for more complex geometries with the bespoke passive internal flow. Furthermore, the proposed fabrication workflow can be adopted at the production level, enabling large-scale manufacturing of inertial microfluidic devices.

## Introduction

Continuous separation of particles and cells is required for a wide variety of applications that include mineral processing, chemical syntheses, environmental assessments, and biological assays^1^. A number of conventional methods exist for this purpose; however, they have several drawbacks. Membrane filtration-based techniques, while efficient and simple, are limited by filter fouling and clogging. Centrifugation methods are also plagued by problems of particle adhesion and clogging, along with their high cost and inability for continuous processing. Likewise, techniques based on sedimentation are prone to particle adhesion and slower processing time, which increases the non-viability of cells in biological applications. Also, methods based on magnetic-activated cell sorting (MACS) and fluorescence-activated cell sorting (FACS) are proven to be low throughput and expensive^2–4^.

With the evolution of microfabrication and rapid prototyping techniques, microfluidic technology has emerged as an alternative to improve upon conventional separation techniques^5,6^. These microfluidic techniques are grounded on the unique characteristics of microscale flow phenomena and have recently gained prominence as efficient tools for the control and focusing of microbeads. Amongst existing microfluidic systems, inertial microfluidics has experienced massive growth in many applications ranging from cell separation^7,8^, cytometry^9,10^, multiplexed bio-assays^11,12^, and also fluid mixing^13^. Despite great advantages of inertial microfluidics, the commercial impact and scalability of this technology have been restricted due to fabrication issues.

As a passive technique, inertial microfluidic systems manipulate cells and particles by taking the advantage of hydrodynamic forces in microchannels with a variety of cross-sections. To date, several microchannels (i.e., straight, spiral, and serpentine) with different cross-sections (i.e., square, rectangular, triangular, trapezoidal, and circular) have been proposed to enhance particle sorting by optimizing the synergetic effects of inertial and Dean drag forces^14–18^. These devices are mainly fabricated by casting PDMS on a master mold, which is made by either standard microfabrication techniques (i.e., silicon etching or SU8 lithography) or using conventional micromilling on an aluminum or polymethylmethacrylate (PMMA) sheets^19–21^. While this approach has been the workhorse behind the development of majority of these devices, the inability to build non-orthogonal and non-planar structures, cost, and labor intensiveness of the process have hampered its widespread applications and commercialization^22,23^.

Besides the aforementioned approach, other groups attempted to develop alternative strategies for the fabrication of inertial microfluidic devices. For instance, several groups reported the usage of femtosecond laser irradiation and CO_2_ laser ablation techniques to produce straight and spiral shape microchannels inside a glass or PMMA^24–28^. Despite the simple fabrication process, the complexity of building non-rectangular cross-sections, poor surface finish, and lengthy etching steps are making them less user-friendly. Some groups also proposed the utilization of metal micro-wires or a sacrificial template in conjunction with softlithography to produce inertial microfluidic devices^29–31^. In spite of the simplicity of this method in the fabrication of circular channels, PDMS rupture or distortion and the presence of residuals in microchannels during the template removal restrict its utility. Recently, the fabrication of PMMA microchannels using hot embossing technique has also been reported. While this method is attractive for rapid prototyping and high volume production of microfluidic systems with microscale features, the necessity of using sophisticated equipment limits its widespread usage^32,33^.

Recently, additive manufacturing has emerged as a powerful platform to fabricate 3D functional microfluidic systems from a variety of polymeric materials^34^. This outstanding technology enables investigators to build microstructures with complex shapes and geometries in a short time^35,36^. Benefiting from the stereolithography apparatus (SLA) technique^37^, Lee *et al*. directly fabricated a 3D helical trapezoidal microchannel to separate *E. coli* bacteria using magnetic nanoparticle clusters^38^. However, due to the poor transparency of the fabricated channel, imaging (whether fluorescent or bright field) was not feasible through the channel. Besides, to remove residuals from channels, the channel width is in the order of millimeter-sized dimensions, which is not suitable for most of the inertial microfluidic applications where small cells or particles are of interest. More lately, 3D printing of sacrificial molds combined with softlithography has gained significant attention due to its simplicity and cost-effectiveness^39^. Gaining the efficiency of the fused deposition modeling (FDM) printer, Tang and colleagues^40^ fabricated various microchannels with unconventional cross-sections to study the effect of geometry on elasto-inertial focusing. While this approach is suitable to fabricate microchannels with different cross-sections, the resolution of printed parts is not high enough due to inherent limitations of FDM printing. Although direct fabrication of microchannels using SLA and digital light processing (DLP) method is a suitable candidate, inertial microfluidic devices often operate in channels in the order of micrometer (e.g., rectangular with 200 µm width and 40 µm height) where removing resin residuals from the channel is a challenging issue^41^.

To address these inadequacies, we have developed a robust protocol for large-scale manufacturing of inertial microfluidic systems. Thanks to the capabilities of DLP and SLA 3D printing^42,43^, we have printed a wide range of microchannels with different geometries, capable of performing particle and cell focusing for various Reynolds numbers (Re). The approach makes the use of a double-coated pressure-sensitive adhesive tape that perfectly binds open 3D-printed microchannels with optically transparent acrylic sheets, producing a leakage-free interface for inertial microfluidic applications. The bonding strength is quantified, and the compatibility of the concept for the fabrication of new generation of inertial microfluidic devices is evaluated using cells and particles.

## Results and discussion

### Fabrication and characterization of 3D-printed channels

Softlithography using PDMS and a master mold is a frequently used method for the fabrication of microfluidic systems. This strategy has several advantages; for instance, PDMS is biocompatible, optically transparent, and gas permeable, which makes it suitable for a myriad of biological applications^44^. Also, 3D printing of PDMS has been reported using stereolithography approach^45^. However, certain drawbacks such as lack of chemical stability, deformation under pressure, and adsorption of small hydrophobic molecules have hindered its industrial-scale utilization. Moreover, the manual molding, cleaning, and bonding process complicate the mass production.

Although theoretically straightforward, scaling up of PDMS-made microchannels for commercialization application in inertial flows is challenging since these devices are flexible and prone to rupture or collapse at high flow rates. In addition, inflation and hysteresis feature of PDMS create a big question mark regarding the exact focusing position of particles. This becomes more serious in CFD modeling where the “fixed wall boundary condition” is not truly correct in inertial regimes within PDMS-made microchannels. It is not surprising that the results of numerical simulations must be validated with hard chips rather than soft (PDMS-made) microchannels. Apart from these issues, the inherent limitations in the standard microfabrication and softlithography techniquesmake researchers unable to explore particle migration in unconventional cross-sections (e.g., right-angled triangular or hexagonal). For instance, particle focusing within a triangular curved microchannel has never been explored due to the fabrication difficulty. As such, there is a great need to develop standardized protocols for the fabrication of inertial microfluidic devices to facilitate ground-breaking research, while enabling quick translation into commercial products.

**Figure 1:**
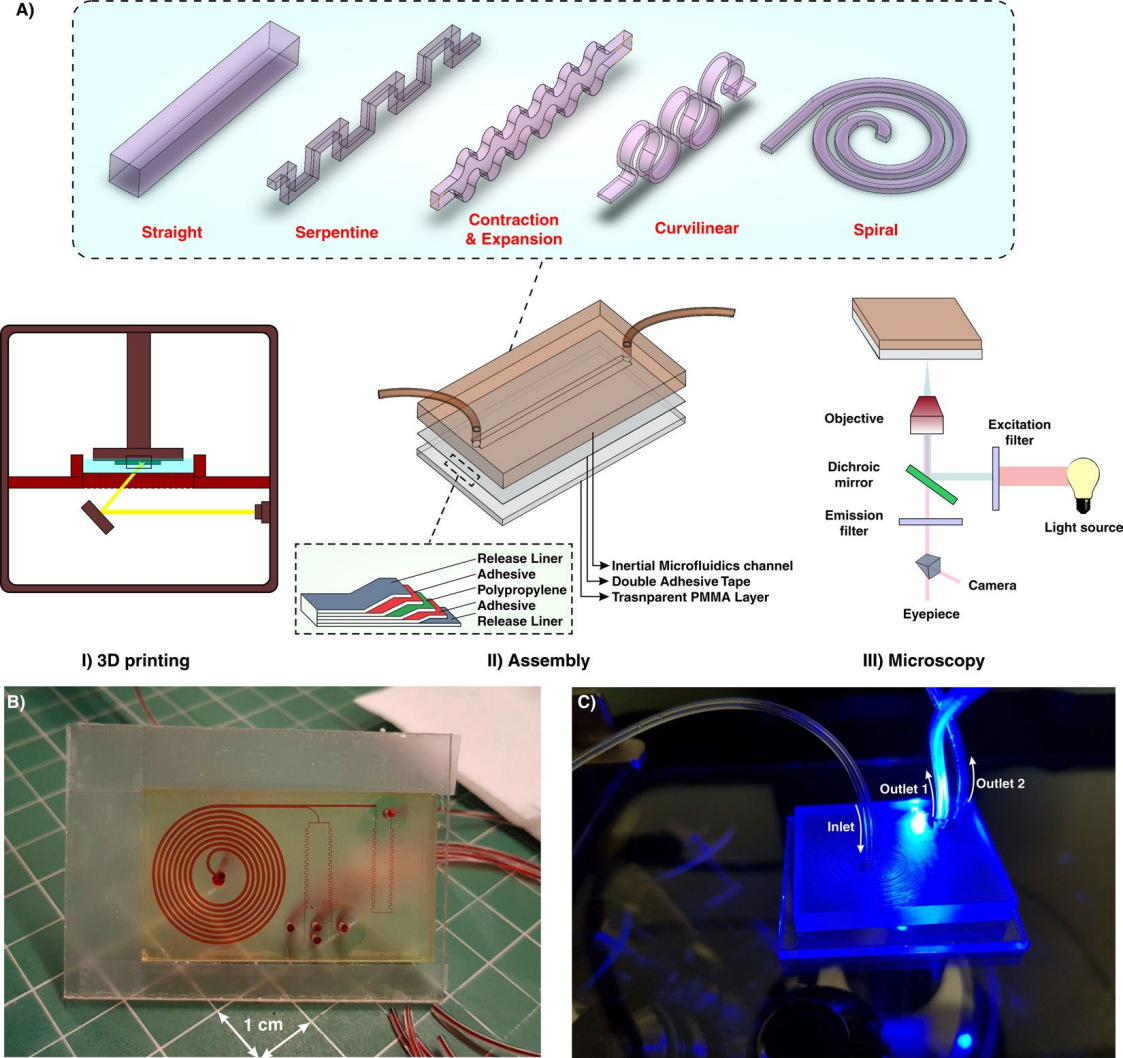
(**A**) Schematic illustration of the proposed workflow for the fabrication of inertial microfluidic devices. I. The desired channel geometry was printed by a high-resolution SLA/DLP 3D printer II. After cleaning the part by isopropanol, it was bonded to a PMMA sheet by means of a double-coated pressure-sensitive adhesive tape. The entire process just takes less than two hours. III. Benefitting from the PMMA transparency, high-speed, fluorescent, bright field, or phase contrast microscopy can be performed from the bottom side of the channel (**B**) An actual complicated inertial microfluidic device containing a spiral and serpentine microchannel. (**C**) Fluorescent microscopy from the bottom side of the channel.

In this study, we have proposed a novel approach for the fabrication of inertial microfluidic devices based on the 3D printing method. Figure 1 demonstrates an overview of the fabrication process. Gaining the efficiency of a high-resolution 3D printer, the desired microchannel is printed while its face (where the design pattern exists) is outer, and the base is attached to the build plate (Fig. 1AI). This method is particularly significant since the change of cross-sections in inertial microfluidics is of great interest. However, the printing parameters need to be optimized for the fabrication of a channel with proper and accurate dimensions. The slice thickness in Z direction, curing time of each layer, and total thickness of the part are the most critical factors to have a high-quality channel with a great surface finish. Various cross-sections, ranging from right-angled or isosceles triangular to hexagonal, were fabricated and the best optimized parameters were identified (Fig. S1). To complete the fluidic network, 3D-printed inertial microchannels need to be bonded to a substrate with enough optical transparency and rigidity for subsequent testing. In this work, a variety of scenarios has been evaluated, and upon extensive evaluations and characterizations, permanent bonding of 3D-printed channels to a PMMA sheet via a double-coated adhesive tape was selected as the most promising and reproducible method. A transparent double-coated pressure-sensitive adhesive tape (ARcare, Adhesive Research) having 25.4 µm clear polyester film coated with AS-110 acrylic medical grade adhesive was cut with a similar size of PMMA sheet (Fig. 1AII). After the attachment of one side of the tape to the PMMA sheet, the 3D-printed inertial part was manually placed over the other side of the tape and pressed with a tweezer until no bubble was observed at the interface (Fig. S2).

An important feature of PDMS is its optical transparency, which makes it suitable for a broad range of microscopic applications. Given the fact that commercial DLP/SLA resins are not typically transparent, the attachment of 3D-printed microchannels to PMMA sheets provides enough transparency for the optical and fluorescent microscopy (Fig. 1AIII). What makes this approach attractive for a wide range of communities (e.g., biologists and chemists) is its user-friendliness for people without prior knowledge about microfabrication and softlithography. The entire process from CAD drawing to printing and then testing takes less than 2 hours, portraying the versatility of this method for inertial microfluidic research. More importantly, devices made using this technique are not prone to the deformation and leakage compared to the PDMS-made devices, making them suitable to study new physics, especially at high Re. Furthermore, by considering the fabrication cost, time, and efforts of a complicated inertial microfluidic device, our suggested method is rapid and utilizes a low-cost raw material which are valuable features, especially in areas where resources are limited. Figure 1B,C depict a final device fabricated using this technique. The internal channels are filled with red food color for the sake of illustration.

**Figure 2:**
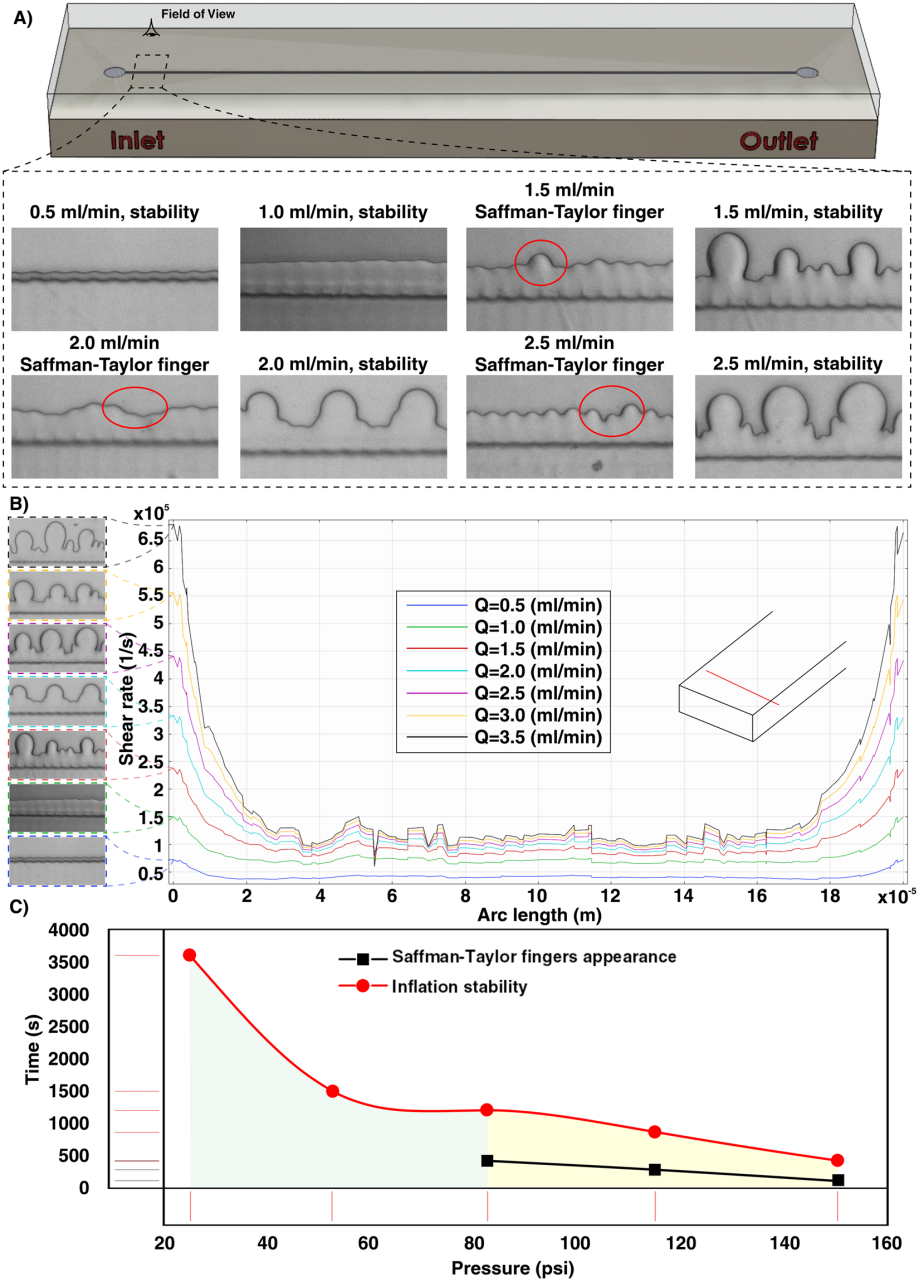
(**A**) Analyzing the Saffman-Taylor finger criteria for the bonding quality in a microchannel versus various flow rates. For flow rates lower than 1.5 ml/min, the Saffman-Taylor fingers do not appear, while for flow rates more than 1.5 ml/min, Saffman-Taylor fingers become discernible. (**B**) Shear rate distribution across a line parallel to the channel width. (**C**) The more the pressure, the faster the creation of Saffman-Taylor fingers. In the green area, Saffman-Taylor fingers do not appear during the experiments. Also, the results show that there is not any evidence of channel burst or delamination during the bonding quality test.

In order to investigate the bonding quality, a straight microchannel with dimensions of 50 µm height, 200 µm width, and 4 cm length was fabricated and tested accordingly. We have monitored the device performance for the appearance and growth of Saffman-Taylor fingers until it becomes stable, called “inflation stability” (Fig. 2A). The results are presented in a 2D diagram to identify the channel behavior at a given pressure, as shown in Fig. 2C. Our results revealed that the holding strength of double-coated adhesive tape was able to achieve a leak-proof interface between the 3D-printed part and PMMA sheet, not only at typical operating pressure reported in literature^46^, but also more than the capability of PDMS-made channels in withstanding high flow rate conditions. Shear rate distribution across a line parallel to the channel width was also evaluated, and as Fig. 2B revealed, increasing the flow rate leads to imposing more shear forces at the edges of the channels. The more the flow rate, the larger the appearance of Saffman-Taylor fingers (insets of Fig. 2B). The green area in Fig. 2C shows the safe zone for performing inertial microfluidic experiments where no Saffman-Taylor fingers appear during the operation. We have found that at pressures more than 82.6 psi, Saffman-Taylor fingers begin to appear; however, this does not impose any detrimental effect on the device performance (i.e., no leakage or bonding collapse). Also, we did not observe any delamination or deformation in channels after consecutive runs at high pressures (i.e., 120 psi), all of which are common in PDMS-based inertial microfluidic devices (see Figs. S3 and S4 for the pressure drop, velocity profile inside the microchannels for a wide range of operating flow rates).

**Figure 3:**
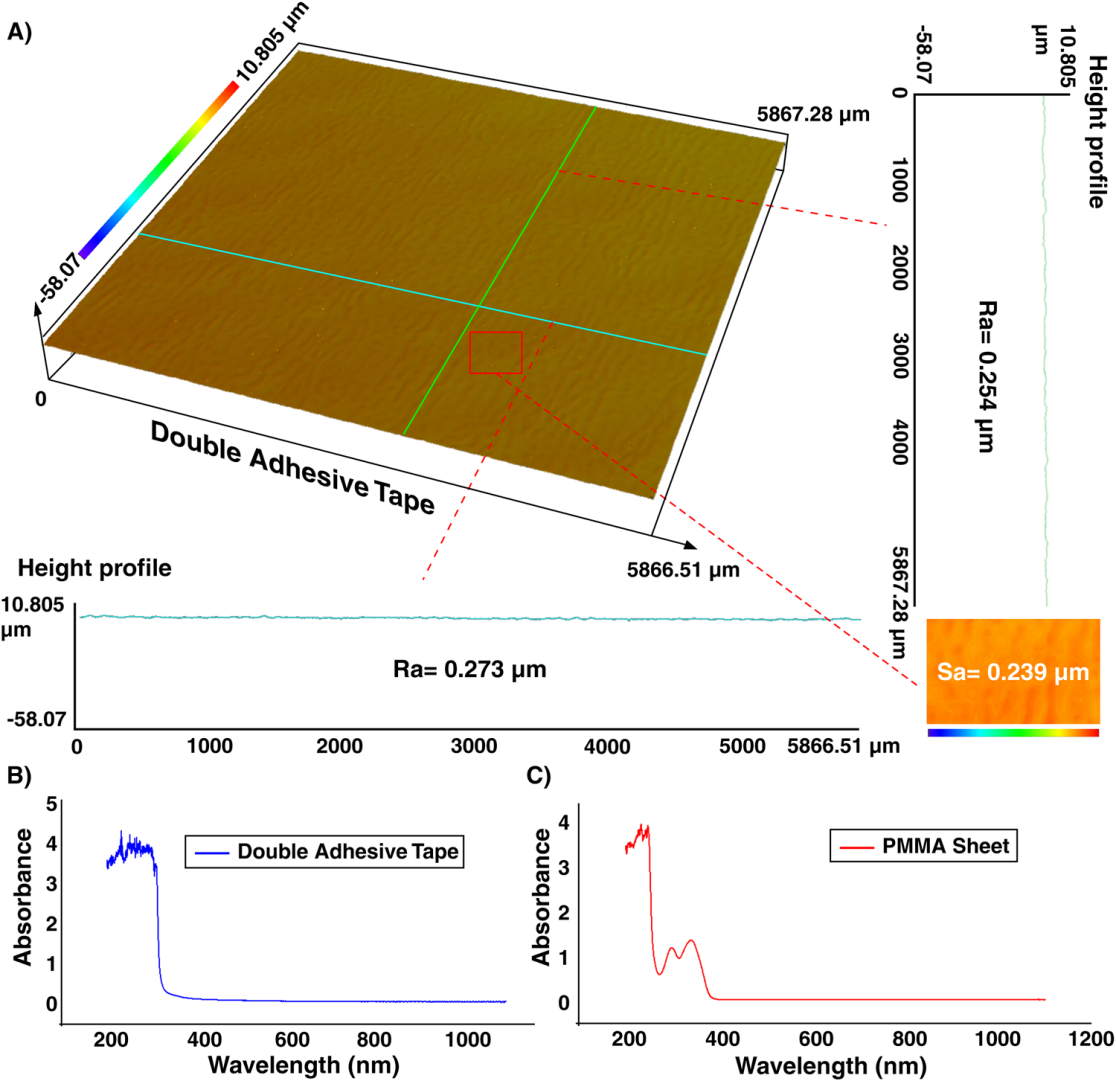
(**A**) Surface topography of the double-coated adhesive tape. Results in a vertical line (green line), horizontal line (blue line), or across an area (red rectangular) show that the tape has homogenous roughness with a nanometric value, which does not impose any interference on the channel performance. The absorbance amount of (**B**) double-coated adhesive tape and (**C**) PMMA sheet, implying that these two materials are transparent for visible spectra range, and there is not any significant absorption.

The surface characteristic of the double-coated adhesive tape was also investigated using a profilometer. As Fig. 3 illustrates, the roughness of the tape is homogenous and is in the submicron range. The values of *Ra* and *Sa* were about 250 and 240 nm, respectively. Also, the roughness of the 3D printed parts was evaluated and value of *Sa* was less than 300 nm. These nanometric rugosities indicate that the roughness of tape does not have any effect on the flow profile and particle focusing. Although optically transparent, the optical characteristics of the PMMA sheet (2-mm-thick) and adhesive tape were evaluated to identify the possibility of accurate fluorescence imaging^47^. Hence, the UV-visible absorbance spectra for a wide range of wavelengths (i.e., from 200 to 1100 nm) were recorded via a spectrophotometer (Cary 60 VU-Vis spectrophotometer, Agilent Technologies). Figure 3B,C reveal that the light loss is negligible for both PMMA and adhesive tape within the visible spectrum, resulting in no trace of autofluorescence residual.

### Straight microchannel

Straight microchannels with rectangular or square cross-sections are arguably the most widely used inertial microfluidic systems. Thanks to their ease of fabrication and the ability for parallelization, a myriad of applications have been developed using these platforms over the past decade^48^. The required channel length for inertial particle migration to the equilibrium positions is ${L}_{f}=\pi \mu {H}^{2}/\rho {U}_{m}{\alpha }^{2}{f}_{L}$ where *f*_*L*_ is estimated in the range of 0.02 to 0.05 for (*H*/*W*) from 2 to 0.5, and the corresponding flow rate for inertial migration is calculated as $Q\approx 2\pi \mu W{H}^{3}/3\rho L{\alpha }^{2}{f}_{L}$^48^. Channel Re (*Re* = *ρUD*/*μ*) and particle Reynolds number $(R{e}_{p}=Re\frac{{\alpha }^{2}}{{H}^{2}})$ are two dimensionless numbers for the characterization of particle migration in a straight microchannel. When particle Re is much smaller than 1, viscous drag becomes dominant, and particles follow the streamline. Increasing particle Re augments inertial forces, causing inertial particle migration become obvious in the microchannel^49,50^.

**Figure 4:**
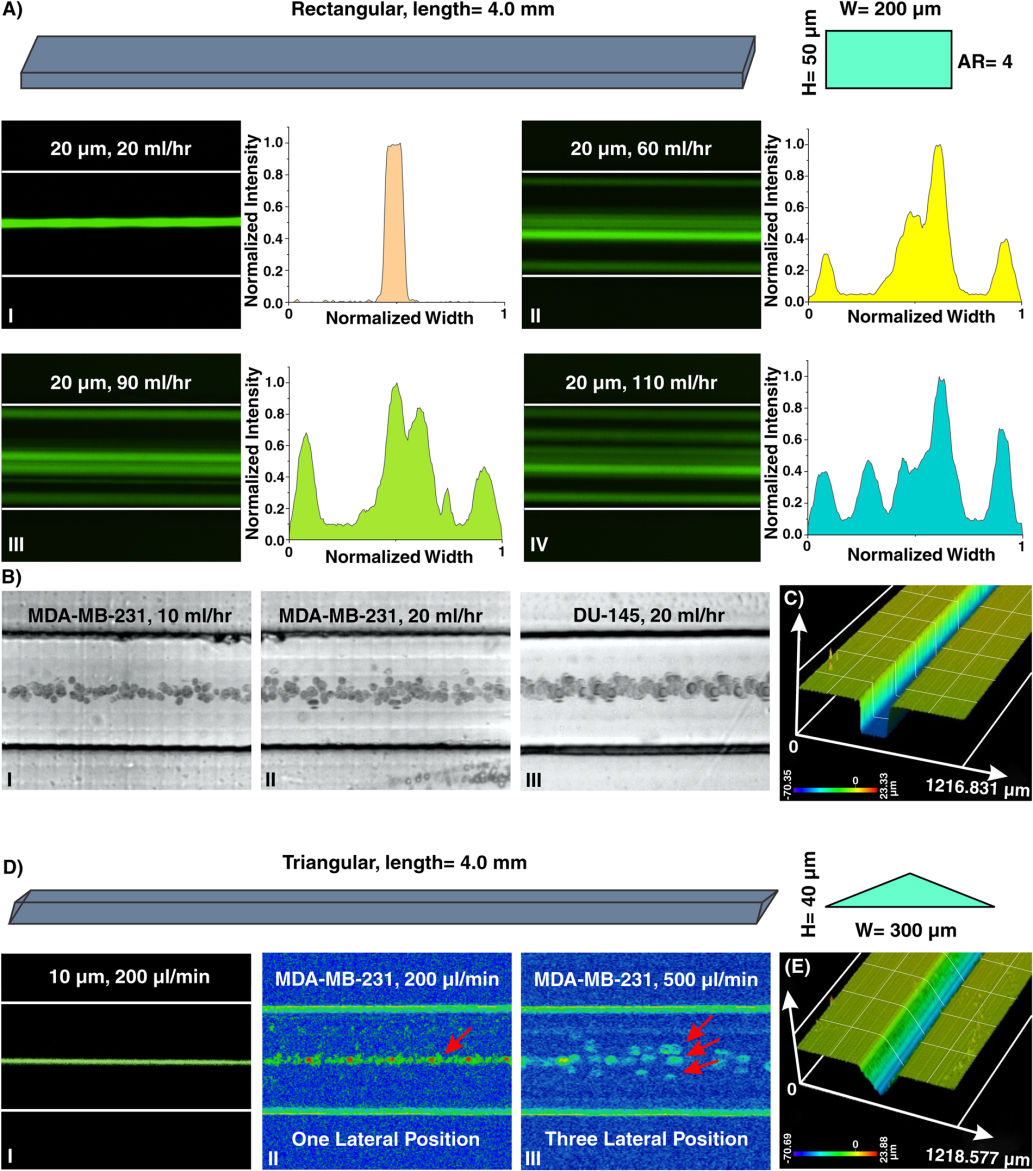
(**A**) Inertial microfluidics in a rectangular straight microchannel with height and width of 50 µm and 200 µm, respectively. I. At first, 20 µm particles occupy the center of the channel as their focusing position. The intensity profile also illustrates that particles are focused at the center of the channel. II–IV. Later, looking at lateral position and intensity profiles reveal that by increasing the flow rate, side walls are added to the focusing position of the particles, and the focusing band of particles at center becomes wider. To extract these images, we have used “max intensity” feature from Fiji Software (https://fiji.sc). (**B**) The equilibrium position of MDA-MB-231 cells at flow rates of I. 10 ml/hr and II. 20 ml/hr and III. DU-145 cells at flow rate of 20 ml/hr confirms the single-line focusing of cells within the rectangular straight microchannel (from top view). (**C**) A surface profilometry of the rectangular cross-section which shows the rectangular profile of the microchannel. Results show that the channel has perfect shape and quality which is suitable for inertial microfluidics. (**D**) In triangular microchannel, particles first migrate to I. and II. one focusing position and then this increases to III. three separate points. This trend is similar to those reported in the literature^14,53,54^. The results for MDA-MB-231 cells at flow rate of 200 µl/min illustrate a single-line focusing position, and at flow rate of 500 µl/min depict three focusing positions. (**E**) Surface profilometry of the triangular straight microchannel with height and width of 40 µm and 300 µm, respectively.

Particle migration within a straight channel strictly depends on its cross-section. In square straight microchannels (with an aspect ratio (AR) (width/height) of 1), particles migrate to four equilibrium positions located at the center of each wall. Changing the cross-section to rectangular disturbs this focusing pattern where in a rectangular straight microchannel with AR of 0.5, focusing positions reduce to two near the center of long walls^51^. This behavior was explained by Zhou and Papautsky where they identified two-stage particle migration in rectangular straight microchannels^21^. Further increase in the AR results in the more unpredicted focusing behavior of particles. Generally, in channels with high AR, stable focusing positions are reduced. However, by exceeding Re from a critical value, the number of stable equilibrium positions increases which is a function of particle size, channel dimensions, and Re. Based on reported experimental results, $R{e}_{c}=697{(AR/\kappa )}^{-0.79}\,(4.5\le AR/\kappa \le 60,\,5\le Re\le 660)$ was identified^52^. The abovementioned results elucidate that particle focusing is strongly affected by channel cross-section. However, due to the fabrication limitations, dependency of various cross-sections to channel geometries was not systematically investigated. Recently, triangular and semi-circular cross-sections were fabricated using Si anisotropic etching with potassium hydroxide^53^, a brass for mold fabrication^54^, FDM for creation of sacrificial mold^40^, or unconventional micromilling^14^. However, critical fabrication limitations do not allow for further investigation on the dependency of triangular angle or type (e.g., right-angled triangular) on focusing patterns of the particles. Here, as a showcase, a straight microchannel with rectangular cross-section and AR of 4 (all channel dimensions are provided in Section S3 and Tables S1–5) was fabricated, and the results are illustrated in Fig. 4A. As the results indicate, for low Re (Fig. 4AI), 20 µm particles focus at the center of long walls of the channel cross-sections, shown previously in PDMS-made microchannels. Nonetheless, the focusing pattern for particles at higher Re does not obey a specific role. As clearly can be seen, increasing flow rates leads to generation of additional focusing positions within the microchannels where side walls are also added to the equilibrium positions of particles (Fig. 4AII–IV). Furthermore, lateral migration of MDA-MB-231 and DU-145 cells at low flow rates (10~20 ml/hr) (Fig. 4BI–III) illustrates their single-line focusing within the rectangular straight microchannel, which is promising for flow cytometry applications. Moreover, to showcase the versatility of the proposed method, a triangular straight microchannel was fabricated and the results are shown in Fig. 4D. The results are completely in line with those reported in the literature where 10 µm particles and cells occupy one lateral position in the channel for low flow rates (Fig. 4DI,II). For high flow rates, this equilibrium position increases to three points which are captured using MDA-MB-231 cancer cells inside the channel (Fig. 4DIII)^14,53,55^. Surface profilometry of the channel cross sections (rectangular and triangular) (Fig. 4C,E) confirms the accuracy of the method for the fabrication of various cross-sections.

### Sinusoidal and serpentine microchannel

**Figure 5:**
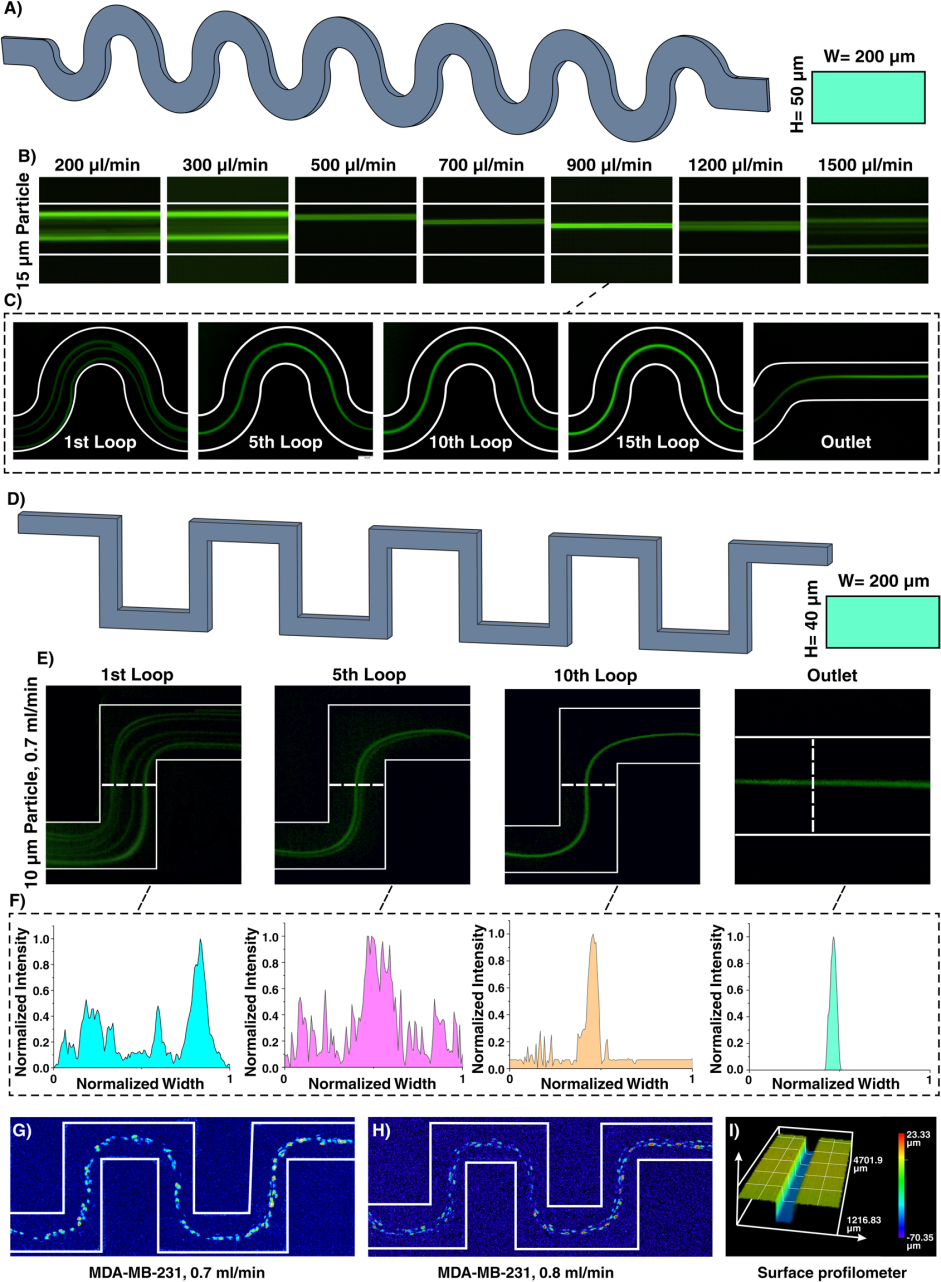
(**A**) Inertial microfluidics in a curvilinear microchannel with height and width of 50 and 200 µm, respectively. (**B**) The results show that the equilibrium position of particles depends on the flow rate and has different focusing modes. Particles first focus at two equilibrium positions and then occupy just one focusing line. Eventually, by further increasing the flow rate, Dean drag forces become dominant, resulting in defocusing of particles. The trend is similar to that reported in the literature^60^. (**C**) 15 µm particle migration throughout the channel for flow rate of 900 µl/min, demonstrating that particles are focused at the 5^th^ loop. (**D**) Inertial microfluidics in a serpentine microchannel with height and width of 40 and 200 µm, respectively. The number of lateral positions depends on the applied flow rate at the entrance of the channel. (**E**) Focusing behavior of 10 µm particles at 0.7 ml/min. (**F**) As intensity profile elucidates, at the 10^th^ loop, particles reach the stable equilibrium position. Lateral migration of MDA-MB-231 cells at flow rate of (**G**) 0.7 ml/min and (**H**) 0.8 ml/min shows a single-line focusing of these cells at the center of the channel. (**I**) Surface profilometry of the channel with rectangular cross-section with width and height of 200 and 50 µm, which shows the accuracy and high-quality of the channels appropriate for inertial microfluidics.

Inertial microfluidics in sinusoidal (curvilinear) microchannel has gained traction due to its improved focusing performance, the ability of massive parallelization, and small footprint. In the sinusoidal microchannel, the curvature direction changes in each loop, resulting in an intricate phenomenon that help in particle focusing. Indeed, by alternating the curvatures, the direction of Dean flows changes, and secondary flows may not reach a steady-state condition. This design was firstly developed by Di Carlo in 2007 and its capability in wide ranges of Dean number $(De=Re\sqrt{{D}_{h}/2R}$, where Re is channel Re, *D*_*h*_ is characteristic length of channel, and R is the radius of channel curvature) was evaluated^56^. By assuming that Dean drag forces were balanced with shear gradient lift forces, his team proposed the ratio of inertial lift forces to Dean drag forces as ${F}_{L}/{F}_{D}=2r{a}^{2}/{D}_{h}^{3}$ (where *r* is radius of channel and *a* is particle diameter). Generally, if *F*_*L*_/*F*_*D*_ ≫ 1, secondary flows do not affect particles, and if *F*_*L*_/*F*_*D*_ ≪ 1, particles are entirely affected by secondary flows^57^. The application of this microchannel was even expanded where it was used for high-throughput separation of micron and sub-micron bioparticles (cyanobacteria)^58^ and a microfluidic concentrator for harvesting of cyanobacteria^59^. In addition, in a comprehensive study, the design principle of curvilinear microchannels was investigated, and a map for various focusing phenomena was provided, based on ${F}_{L}/{F}_{D} \sim (R{e}^{2}/D{e}^{2}){(a/{D}_{h})}^{3}{f}_{L}$ where *f*_*L*_ was approximated by Zhou and Papautsky^21^ as *f*_*L*_ ~ 1/*Re*(*D*_*h*_/*a*)^2^ ^60^. The dependency of curvature angle^61^ and various cell lines^62^ on the focusing positions was also evaluated. In order to showcase the adaptability of our method for fabrication of various inertial microfluidic devices, a curvilinear microchannel with rectangular cross-section (Fig. 5A) was designed, fabricated, and evaluated. Figure 5B reveals that 15 µm particles first occupied two focusing positions and by increasing the flow rate, it reduced to a single focusing line across the channel (e.g., at 5^th^ loop for flow rate of 900 µl/min (Fig. 5C)) which is consistent with the previously reported results^60^.

By altering the design of curvilinear to straight, serpentine microchannel with a square-wave pattern is created. This channel proves to have unique features for size-based particle focusing. Generally, particle focusing achieves when *a*/*D*_*h*_ > 0.07 and *R*_*p*_ ~ 1. However, additional secondary flows in serpentine microchannel lead to focusing of particles with smaller diameters compared to those calculated by the above formula. These devices can benefit from parallelization along their vertical direction, thereby increasing their throughput. Three focusing patterns can be identified by increasing the input flow rate, i.e., two-sided focusing, transition focusing, and central single-line focusing. If inertial effects dominate the secondary flows, particles occupy two lines near the walls. In contrast, dominance of secondary flows results in a single-line focusing at channel center. If these two effects have the same order, particles focus as a wide streak. Gaining the efficiency of two-sided focusing for small particles and central focusing for big ones give us the opportunity of size-based particle separation. Based on the literature, a serpentine microchannel with cross-section of 40 × 200 µm (*H* × *W*) and 15 loops was fabricated and used to showcase the focusing of 10 µm particles (Fig. 5D).

In a straight channel with 40 × 200 µm (*H* × *W*) cross-section, *a*/*D*_*h*_ for 10 µm is 0.056, which is less than the focusing criteria (0.07); theoreically, these particles cannot focus in a straight microchannel. However, in the serpentine microchannel with the aid of secondary flows, 10 µm particles can efficiently be focused. Figure 5E elucidates that 10 µm particles at flow rate of 0.7 ml/min can be focused at the center of channel at the 10^th^ loop and occupy central equilibrium position at the outlet, which is consistent with the insets as normalized intensity profile of the particles (Fig. 5F). More importantly, the performance of device was tested with MDA-MB-231 cells (Fig. 5G,H), and the results are consistent with those reported in the literature^63^. The surface profilometry of the channel cross-section is also provided in Fig. 5I, indicating the high-accuracy of the proposed method for fabrication of inertial microfluidic devices.

### Spiral microchannel

**Figure 6:**
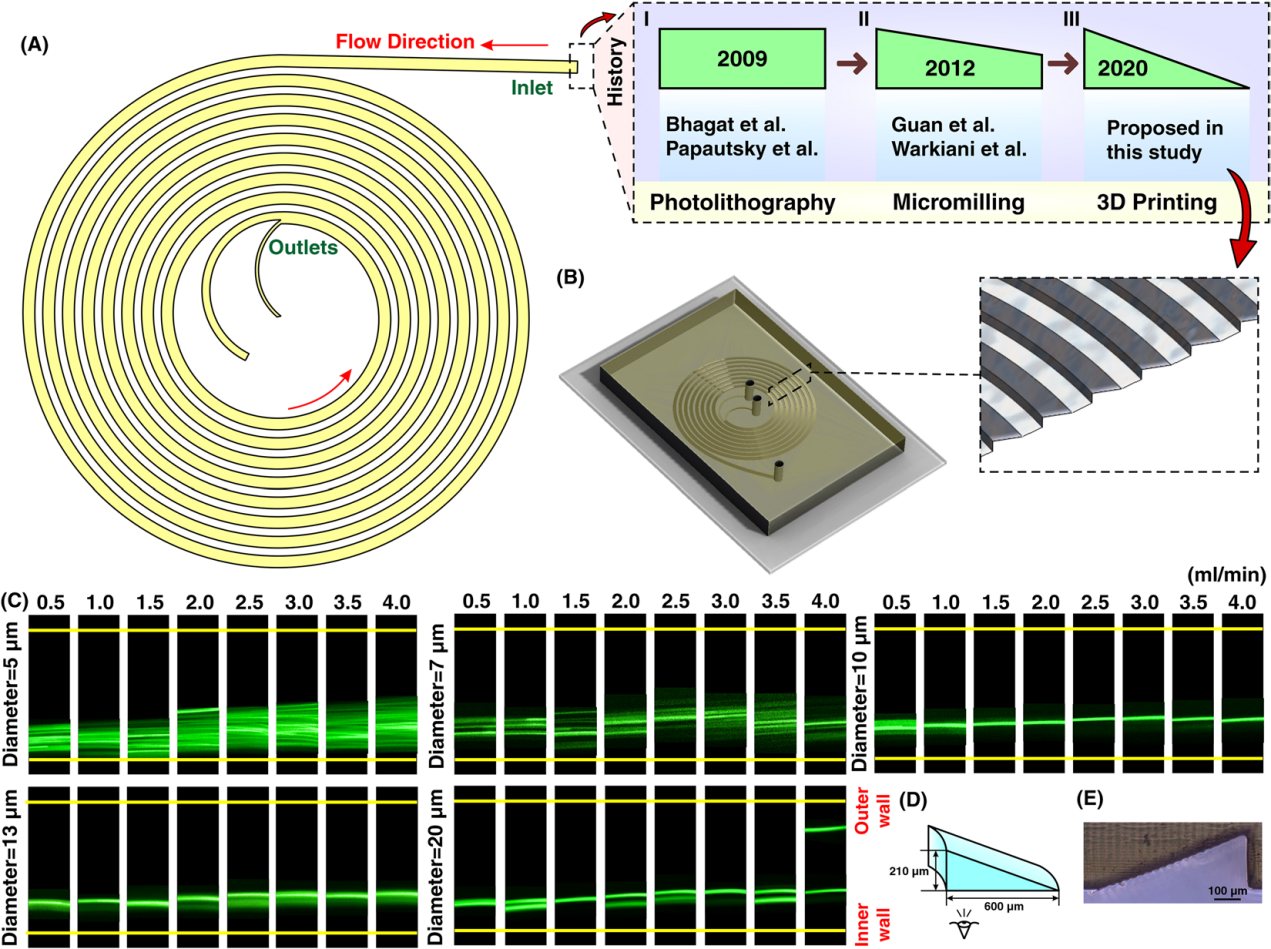
(**A**) Illustration of a spiral microchannel where the fluid direction is from outside to inside. I. Firstly, several groups (e.g., Bhagat *et al*.^9^, Papautsky *et al*.^66,77^, etc.) have shown the capability of rectangular spiral microfluidics for such applications as flow cytometry or microparticle/cell separation. The fabrication of these devices was based on photolithography. II. Gaining the efficiency of micromilling, many groups (e.g., Guan *et al*.^67^, Warkiani *et al*.^20^, etc.) made an attempt to get the advantage of trapezoidal spiral microchannel for particle/cell filtration and fractionation. III. In this study, for the first time, we have shown the fabrication of a right-angled triangular spiral microchannel with the aid of additive manufacturing. (**B**) Schematic illustration of the microchannel where the inset shows the cross-section of the microchannel. (**C**) Results reveal that for particles larger than 10 µm, a tight focusing band appears at the outlet of the channel. Also, for larger particles at high flow rates (i.e., 4 ml/min) double-band focusing appears. (**D**) dimensions of the right-angled triangular spiral microchannel where the inner wall is 210 µm and the width is 600 µm. The hydraulic diameter of this channel is similar to a spiral with a trapezoidal cross-section and the dimension of 80 × 130 × 600 µm. (**E**) Illustration of a right-angled triangular cross-section, which shows the accuracy of the fabrication process.

Spiral defines as a curve winding around a center point with continuous decreasing or increasing manner. When flow passes the curvature, velocity mismatch occurs in the curve section of the channel, resulting in the generation of secondary flows. In inertial microfluidics, spiral microchannel has progressed significantly, and nowadays, most of the particle/cell separations are performed using these microchannels^64^. De is used for the characterization of secondary flows within the channel. Intuitively, smaller channel curvature or larger channel size or Re leads to higher De, thereby imposing stronger secondary flows within the channel. For a given De, average transverse Dean velocity (*U*_*De*_ = 1.84 × 10^−4^ *De*^1.63^) and Dean drag force $({F}_{D}=3\pi \mu {U}_{De}\alpha =5.4\times {10}^{-4}\pi \mu D{e}^{1.63}\alpha )$ can be identified. However, the exact behavior of particle migration at the downstream of the fluid was not thoroughly investigated, and all results are based on experimental data. The most appealing feature of spiral inertial microfluidics is its high-throughput where 2100 particles per second can be processed^9^. Particle sorting is one of the most significant applications of spiral microfluidics. Previously, the potential of a rectangular spiral microchannel for continuous and simultaneous isolation of 10, 15, and 20 µm based on softlithography was investigated (Fig. 6AI)^65^. Dean flow dynamics for a low-aspect-ratio rectangular spiral microchannel was also thoroughly explored^66^. Beyond a simple rectangular spiral microchannel, various geometry modifications for regulation of Dean forces and performance enhancement of the device have been proposed. Benefitting from micromilling (Fig. 6AII), trapezoidal spiral microchannels illustrate promising results in redistribution of lateral focusing positions of particles appropriate for size-based particle separation. In these channels, smaller particles focus along the outer wall, whereas larger ones migrate toward the inner wall^67^. This superior advantage has been widely investigated by our group, among other groups, for circulating tumor cell (CTC) and circulating fetal trophoblasts (CFT) isolation^19,68^, blood plasma separation^69^, isolation of microcarriers from mesenchymal stem cells^70,71^, microalgae separation^72^, and synchronizing *C. elegans*^73^. Also, multiplexing using stack of attached PDMS layers to boost the throughput is illustrated previously^69,74^. However, most of the aforementioned applications are just doable by utilizing cleanroom facilities or employing conventional micromachining (e.g., metal machining or laser cutting) for the fabrication of microchannel. Besides, micromachining has its own limitations such as inability to make sharp corners or difficulty in making spiral loops close to each other. These challenges highlight an unmet need for the fabrication of spiral microchannels using a versatile method which is robust and can surmount aforementioned issues.

As a showcase of the versatility of our proposed method, we have fabricated a spiral microchannel with trapezoidal cross-section with a width of 600 µm and heights of 80 and 130 µm. These results are then put aside a PDMS chip with similar dimensions, and the data is provided in ESI (Fig. S5). Despite all progress in spiral inertial microfluidics, there is not any report of a spiral with cross-sections rather than rectangular or trapezoidal. In other words, a huge amount of potential remains intact to study spiral microchannels with different cross-sections such as triangular (Fig. 6AIII). For this aim, for the first time, we have fabricated a spiral microchannel with right-angled triangular cross-section (as schematically shown in Fig. 6B) where the width and height are 600 and 210 µm, respectively. As the results are illustrated in Fig. 6C, there is a tight band focusing for particles larger than 10 µm, which is suitable for high throughput flow cytometry applications where single line focusing is desired. Also, we observed double-band focusing behavior for 20 µm particles at flow rate of ≥4 ml/min. The dimensions (Fig. 6D) and channel cross-section (Fig. 6E) show the accuracy of the proposed method the for fabrication of right-angled triangular spiral microchannel (check Fig. S6 for contraction-expansion array microchannel results). These results illustrate the flexibility of this method where a complex cross-section can be fabricated in less than two hours with high robustness and stability. Our results hold promise for leveraging the potential of additive manufacturing for the fabrication of inertial microfluidic devices, which is more challenging using conventional microfabrication methods (see Section S6 for multiplexing of 3D printed inertial microfluidic devices).

### Cellular studies

**Figure 7:**
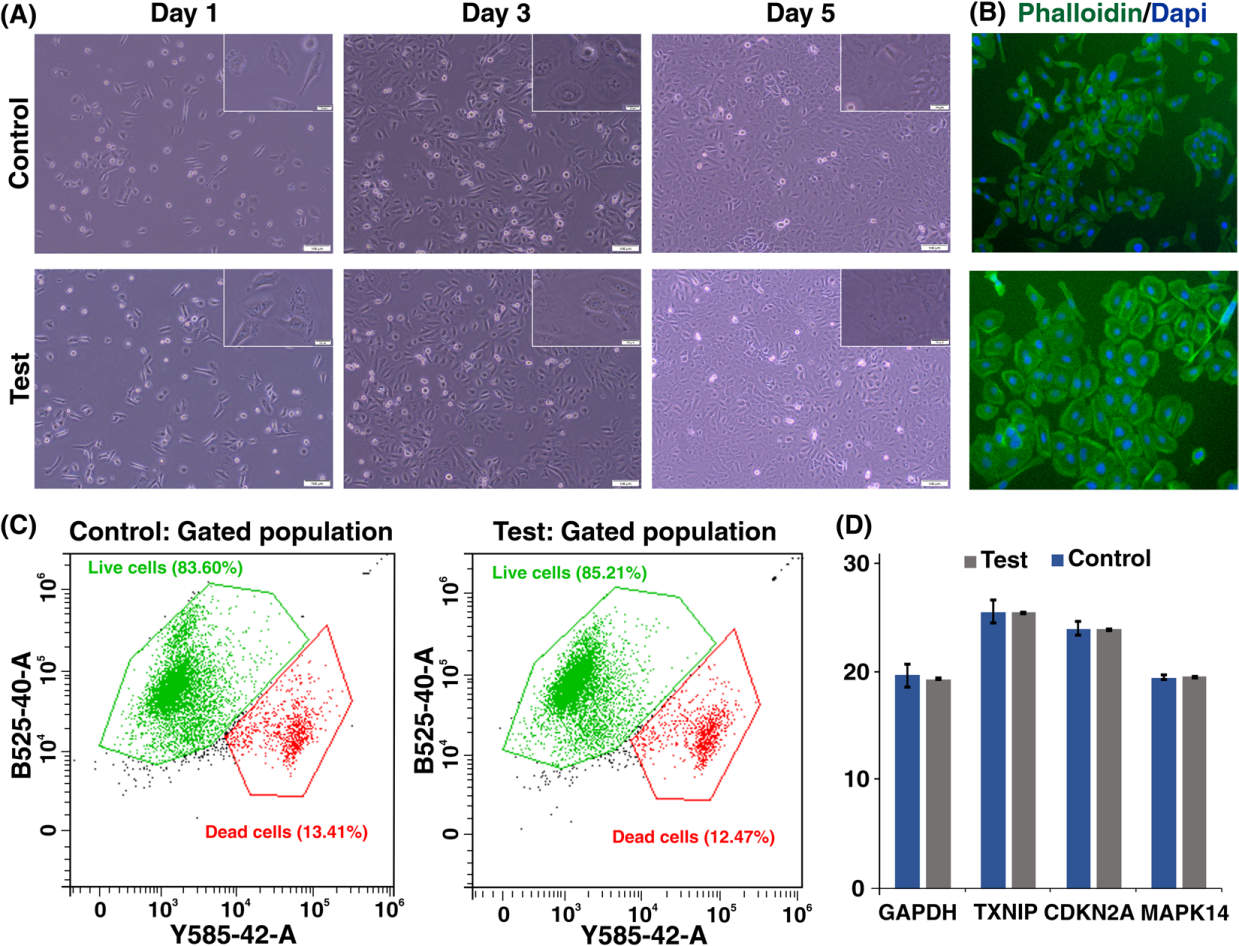
(**A**) Monitoring the morphological feature of cells during five days post-experiment, compared to the control group. (**B**) Fluorescent staining of F-actin filaments in expanded cells on day 5 (green = phalloidin-FITC, blue = nucleus) (**C**) Representative plot and mean value ± SEM of flow cytometric analysis of live/dead population for control and test group. (**D**) Cycle of threshold (Ct) value (expression level) for *GAPDH* (cellular metabolism related gene), *CDKN2A* (cell cycle regulatory gene), *TXNIP*, and *MAPK14* (genes involved in cellular stress response). Results are expressed as mean value ± SEM from three independent experiments. Scale bars are equal to 100 µm in large images and 20 µm in inset ones.

PDMS-made inertial microfluidic devices have been widely used for the cell separation using biological samples such as blood and urine. While PDMS is proven to be a biocompatible material with minimum side effects on cells, we have tested the 3D printed devices using DU145 cells, assessing their viability and functionality post-separation. The collected cells from the device outlet were cultured back into a petri dish for 5 days, showing similar morphological features to the control group as shown in Fig. 7A,B. The flow cytometry tests (Fig. 7C) showed that the viability of the cells was not compromised during the operation using 3D printed devices. The real-time PCR analysis was utilised to assess the expression of genes related to the general activities and stress responses in both treated and untreated cells (Fig. 7D). The similar expression level of *GAPDH* and *CDKN2A* confirmed that neither cellular metabolism nor cell cycle progression were affected after processing through the microchannels. Also, there are no significant changes in the expression of *TXNIP* and *MAPK14*, which are two well-known regulators of cellular stress. In all, the 3D-printed inertial microfluidic device does not alter the cell activity and is safe to be used in biological assays.

## Conclusion

In this study, we have showcased a robust and versatile workflow for the manufacturing of inertial microfluidic devices for both laboratory experiments and industrial applications. The proposed method involves 3D-direct printing of a channel with the desired structure and geometry via a high-resolution DLP/SLA 3D printer. Our approach relies on the bonding of 3D-printed devices (i.e., with high-quality surface finish) to a transparent PMMA sheet via a double-coated pressure-sensitive adhesive tape. Given the great transparency of PMMA layer, these devices can be utilised in bright field, phase contrast, fluorescent, or high-speed microscopy. The bonding quality was evaluated by Saffman-Taylor finger criterion, and the results showed that the device is capable of withstanding pressure as high as 150 psi (nearly triple of the value reported for PDMS). The versatility of this method allowed us to fabricate and evaluate a plethora of existing inertial microfluidic devices as exemplified for fabrication of straight, spiral, serpentine, curvilinear, and contraction-expansion arrays microchannels. The total time frame, from designing a part to starting an experiment, takes less than two hours, allowing multiple experiments in a single day. Also, for the first time, we have fabricated and examined a new inertial microfluidic device, i.e., spiral microchannel with right-angled triangular cross-section which is theoretically impossible to fabricate using photolithography. We believe that the proposed workflow will provide inertial microfluidic devices within the reach of any research groups involving in particle/cell manipulation without strong microfabrication background.

## Materials and methods

### Fabrication method

In this study, inertial microfluidic devices were fabricated using a high-resolution DLP/SLA 3D printer (Ultra 50, Miicraft, Hsinchu, Taiwan) featuring 30 µm XY resolution and 32 × 57 × 120 mm^3^ printing area. The desired inertial microfluidic device is first drafted using a commercial CAD drawing software (SolidWorks 2016) and then translated into STL format, a suitable file for 3D printer language. The file is then sliced in Z direction using the Miicraft software (Version 4.01, Miicraft). The slicing in Z direction (slice thickness) can be adjusted from 5 to 200 µm with an increment of 5 µm. The slicing option is related to the complexity of the geometry. In geometries with ramp or step, the slice thickness should be small, whereas for planar or orthogonal structures, higher slice thickness is suitable. The detailed dependencies of printing parameters are provided in electronic supplementary information (ESI) (Section S1 and Fig. S1). The sliced file is then sent to the 3D printer with UV wavelength of 358–405 nm. The UV light is projected from the bottom of the resin bath (filled with BV-007 resin) and passes through a transparent Teflon film. BV-007 is an acrylate-based resin containing 80–95% acrylate components and 10–15% photoinitiator and additives. Once the UV light cures one layer, the Z-stepper motor moves one slice upward, and the next layer starts to be polymerized. This process continues until the part is printed, successfully. When the part is removed from the picker, it should be rinsed and washed with isopropanol, thoroughly and air-dried by an air nozzle. Afterward, the microchannel is exposed to a UV light with 405 ± 5 nm wavelength within a curing chamber for post curing process. One superior advantage of this method compared to the softlithography is that it does not require punching the holes for inlets and outlets since those are printed as a one-body part by the 3D printer. Eventually, tubes (Tygon tubing, inner diameter: 0.020″, outer diameter: 0.060″) were connected to the microchannel by a tweezer.

### Preparation of bead suspension

Fluorescent microbeads (Fluoresbrite Microspheres, Polysciences Inc, Singapore) with 0.01% volume fraction and various diameters were added to the MACS buffer. The primary usage of MACS buffer is to prevent nonspecific adhesion of microbeads to the tubing of the microchannel. The distribution of particles is illustrated using standard deviation, minimum, or maximum light intensity plots, as reported previously^54^.

### Bonding quality test

Inertial devices are operated at high flow rates; hence, the bonding technique must provide enough strength to prevent leakage from the interface. To evaluate the bonding quality of our proposed technique, a simple 3D-printed straight channel featuring 50 µm height, 200 µm width, and 4 cm length was bonded to a 2-mm-thick PMMA layer. A high-pressure syringe pump (Chemyx Fusion 4000, Chemyx, TX, USA) was used to inject fluids inside the channel from a small syringe (6 ml). Increasing the flow rate leads to the generation of Saffman-Taylor fingers around the inlet, in which the most pressure in the channel present (Section S2). Saffman-Taylor fingers are generated by the movement of a viscous fluid within a porous material^75,76^. As the bonded adhesive tape forms a porous zone between the connecting parts, this theory is applicable for the bonding evaluation. An increase in the applied pressure leads to developments of the Saffman-Taylor fingers until the bonding fails. A CCD camera (DP80, Olympus, Tokyo, Japan) mounted on an inverted microscope (IX73, Olympus, Tokyo, Japan) was used for monitoring the bonding integrity. All recorded data were obtained immediately after the bonding of the 3D-printed channels to a PMMA sheet.

### Surface characterization

For the surface characterization of double-coated adhesive tape, a 3D laser microscope (Olympus LEXT OLS5000) was used, and an LMPLFLN 20x LEXT objective lens (Olympus) was selected. Arithmetic mean deviation (*Ra*), the arithmetic mean of absolute ordinate Z (x, y) documented across a line, and arithmetical mean height (*Sa*), the arithmetic mean of the absolute ordinate Z (x, y) recorded across a region were chosen to evaluate the surface characterization of the tape.

### Cell culture, harvesting, and device operation

DU145 cells (human prostate cancer cell line) were cultured and expanded under standard culture condition (37 °C and 5% CO_2_) using Roswell Park Memorial Institute medium (RPMI, ThermoFisher) supplemented with 10% fetal bovine serum (FBS, Gibco) and 1% Penicillin Streptomycin (Pen/Strp, Gibco). Cells were harvested when the flask was 80% confluence. To obtain a homogenous cell suspension without cell clumps, a sufficient volume of TryplE (Gibco) was added to cover the whole flask, and the flask was incubated at 37 °C for 5 min. The cells were then collected in a 15 ml falcon tube and counted with a hemocytometer. TryplE was then replaced with phosphate buffer saline (PBS, Gibco), and cells were diluted to 10^6^ cells/ml concentration by PBS. Afterward, cells were introduced to 3D-printed microchannels (straight rectangular microchannel with a length of 4 cm, width of 200 µm, and height of 50 µm) at the flow rate of 0.3 ml/min. A group of untested cells was kept as control.

### Morphological analysis and cell viability assay

In order to evaluate the viability of cells after passing through the channels, a live-dead assay was performed using Live and Dead Cell Assay kit (Abcam, Cambridge, UK) after the test. One group of collected DU145 cell suspension was diluted to 5 × 10^5^ cell/ml and incubated with the staining solution for 10 min under room temperature, based on the kit manufacturer’s instruction. Then, the stained cells were processed through flow cytometry (Olympus CKX53, Tokyo, Japan) and analyzed by CytExpert software (Beckman Coulter, Inc.). Live and dead cells were detected by green (λ excite/emit = 488/515 nm) and red (λ excite/emit = 488/617 nm) fluorescent, respectively. Another group of DU145 cells was cultured in a culture flask and stained by live and dead staining after 24 hours without detaching by TryplE. Fluorescent microscopic imaging was taken for adhered cells to identify live cells (with green cytoplasm) from dead ones (with red nucleus) (Fig. S8). The morphology of the attached cells was monitored under an inverted microscope for up to five days. Furthermore, the morphological features of both test and control groups were visualized by fluorescence staining of cytoskeleton after three days. The F-actin filaments were fixed and permeabilized by 4% paraformaldehyde (PFA, Sigma) and 0.2% Triton X-100 (Sigma) and then labeled by Phalloidin- FITC (Sigma).

### Real-time PCR analysis

The effect of shear stress on the expression of genes related to proliferation and survival was measured by real-time PCR (BioRad CFX 96 thermocycler). Briefly, the processed cells were reseeded on a culture dish and kept under standard culture conditions for one day. Next, total RNA of cells were extracted by using PureLink RNA Mini Kit (ThermoFisher), and cDNA was synthesized by applying Revert Aid First Strand cDNA Synthesis Kit (ThermoFisher). Real-time PCR was performed using specific TaqMan primer sets and TaqMan PCR Master Mix (ThermoFisher) with following cyclic conditions: 95 °C for 10 min, followed by 40 cycles of 95 °C for 10 s, 60 °C for 1 min, and 72 °C for 10 s.

## Supporting Information

Supporting Information.
