# Supplementary material for: 3D Printing of Inertial Microfluidic Devices
Source: Sci Rep. 2020 Apr 3;10:5929. doi: 10.1038/s41598-020-62569-9 (PMC7125121; doi:10.1038/s41598-020-62569-9)
Supplement: Supplementary file 1 — Supporting Information. [file 41598_2020_62569_MOESM1_ESM.docx]

**3D Printing of Inertial Microfluidic Devices**

Sajad Razavi Bazaz^1,3¥^, Omid Rouhi^1¥^, Mohammad Amin Raoufi^1,2^, Fatemeh Ejeian^1^, Mohsen Asadnia^2^, Dayong Jin^3,4^, Majid Ebrahimi Warkiani^1,3,4,5*^

^1^School of Biomedical Engineering, University of Technology Sydney, Sydney, NSW 2007, Australia

^2^School of Engineering, Macquarie University, Sydney, NSW 2109, Australia

^3^Institute for Biomedical Materials & Devices (IBMD), Faculty of Science, University of Technology Sydney, Sydney, NSW 2007, Australia

^4^SUStech-UTS joint Research Centre for Biomedical Materials & Devices, Southern University of Science and Technology, Shenzhen 518055, P.R. China

^5^Institute of Molecular Medicine, Sechenov University, Moscow, 119991, Russia

^¥^These authors contributed equally as first author

The detailed description of sections listed below is provided in this part.

- Parameter setting for accurate cross-section fabrication
- Velocity and pressure distribution inside the channel
- Channel dimensions
- Comparison of PDMS-made and 3D printed spiral microchannel
- Contraction-expansion array microchannel
- Multiplexing

## Parameter setting for accurate cross-section fabrication

DLP/SLA 3D printing is being studied in the areas of microfluidic because of their low surface roughness and high accuracy. Printing parameters (which are listed below) are the most important factor affecting the quality of a 3D-printed part.

- Slice thickness
- Total thickness of the part
- Curing time of each layer
- Gap adjustment
- Base layer
- Buffer layer
- Base curing time
- Printing speed

Among all parameters listed above, gap adjustment, buffer layer, base layer, and base curing time are related to the attachment of the part to the picker of 3D printer and mostly prevent failing the part during the printing process. Their descriptions are provided elsewhere [1], and we have kept these parameters constant during the experiment. Also, printing speed was set to slow to increase the surface quality of the parts. Our experiments show that slice thickness, total thickness of the part, and curing time of each layer have the most influence on the accuracy of the channel cross-section. Various cross-sections have been evaluated, and the most optimized parameters are selected based on Fig. S1. As results in Fig. S1 show and surface profilometry of channel cross-sections reveal, by choosing the optimized parameters, the channel cross-section has high degree of quality. Also, the step by step fabrication process, from printing a part to bonding to a PMMA sheet is illustrated in Fig. S2.

## Velocity and pressure distribution inside the channel

In order to gain better insight through fluid behavior inside the channel, a commercially available computational fluid dynamics software, Comsol 5.3a, is used. To this end, a rectangular straight channel with length, width, and height of 4 cm, 200 µm, and 50 µm was considered. Stationary laminar flow was applied to the domain and Navier-Stokes $(\rho\left( \boldsymbol{u.}\nabla\right)\boldsymbol{u=}\nabla.\left[ -p\mathbf{I}+\mu\left( \nabla\boldsymbol{u}+\left( \nabla\boldsymbol{u} \right)^{T} \right) \right]+\mathbf{F)}$ and continuity $(\rho\nabla.\left( \boldsymbol{u} \right)\boldsymbol{=}0)$ equations were solved simultaneously. Here, $\rho$ is the density, $\mu$ is the dynamic viscosity, $p$ is the pressure, and $\boldsymbol{u}$ is the velocity. Dynamic viscosity and density were considered the same as that of the water. Normal inflow velocity was set to the inlet for flow rates ranging from 0.5 to 3.5 ml/min while zero pressure with suppress backflow was applied to the outlet. Incompressible flow without any turbulence model type was set to the physical model. The amount of pressure and velocity were extracted from a line passed through the center of the channel cross-section (Fig. S3 and S4). Based on Fig. S3, the more the flow rate, the more the pressure applied at the channel. Fig. S3 also reveals that since the applied pressure at the inlet is more than that at the outlet, the chance of Saffman-Taylor finger appearance is more at the inlet rather than outlet. Therefore, we have monitored the channel from a region near the inlet of the channel.

## Channel dimensions

The design specifications of all geometries fabricated in this study are provided in Table S1-5.

## Comparison of PDMS-made and 3D printed spiral microchannel

As a showcase of the versatility of our proposed method, we have fabricated a spiral microchannel with a trapezoidal cross-section with a width of 600 µm and heights of 80 and 130 µm. As shown in Fig. S5, particles with a diameter of 10 and 15 µm were tested, and it is shown that the possibility of particle fractionation based on their size exists. These results are then put aside the PDMS chip with the same dimensions. The comparison reveals a weird behavior in hard chips. The presence of a tight double band focusing for particle size of 10 and 15 µm was not previously reported for the PDMS microchips. The nature of PDMS is soft, resulting in inflammation at the presence of flow rate, and this becomes more pronounce at high flow rates. Thus, we hypothesize that this behavior is related to the wall shear effect which is stable in hard and inflates in soft chips. Further study on the inertial focusing of various particles in hard chips while considering the effect of rigidity on lateral particle motion is necessary to validate this hypothesis, which is under development in our group. Currently, fundamentals of inertial microfluidics in hard chips are under investigation; these studies reveal that the theories developed so far for PDMS-based microchannels are not necessarily universal, and hard chips obey different roles and are valid for different ranges of Re.

## Contraction-expansion array microchannel

Deformation of a straight channel by adding and subtracting a specific design creates the so-called geometry of contraction-expansion (CE) arrays microchannels. The CE array can be on one side or double side. Particles with small size migrate in areas near the wall while bigger particles focus at the center of the channel. In these array-based microchannels, pairs of helical vortices and secondary flows developed in the expansion section of the channel due to the abrupt change in cross-section, altering the lateral migration of particles. For larger particles, inertial forces are dominant, and particles are focused at the channel centerline. On the other hand, for smaller particles, the dominant force is secondary flows, which creates a new focusing zone near the sidewalls of the channel [2]. This mechanism was used for separation of Malaria disease [3], plasma from whole blood [4], CTCs [5], and various particle sizes [2]. The geometry of contraction-expansion arrays used in this study was adopted from Jiang and co-workers [6] where the detailed channel description has been provided in Table S4. Particle size of 7 and 15 µm are examined, and the results are shown in Fig. S6. Based on above-mentioned theories, smaller particles tend to occupy near the side walls while larger particles inclined to migrate near the center of the microchannel. Altogether, the proposed method enabled the rapid fabrication of inertial microfluidic devices to establish a novel developed method for these studies.

## Multiplexing

While inertial microfluidic devices are proven to be high-throughput, several applications, including bioprocessing or environmental monitoring, exist where the sample volume is in the order of liter rather than milliliter. Multiplexing of PDMS-made inertial microfluidic devices has been previously reported by our group for the processing of large sample volumes [7, 8]. To showcase the possibility of this scenario using 3D printed devices, we have developed a multiplexed spiral microchannel capable of fluid handling at a flow rate as high as ~ 100 ml/min by stacking six spiral microchannels, as shown in Fig. S7A. The beauty of our proposed workflow is that it does not require plasma bonding of PDMS layers where a misalignment in one layer may impose unfavorable effects on the total functionality of the device. The bottom and side view of the multiplexed channels are illustrated in Fig. S7B and S7C. Channels are filled with a red dye for better illustration.


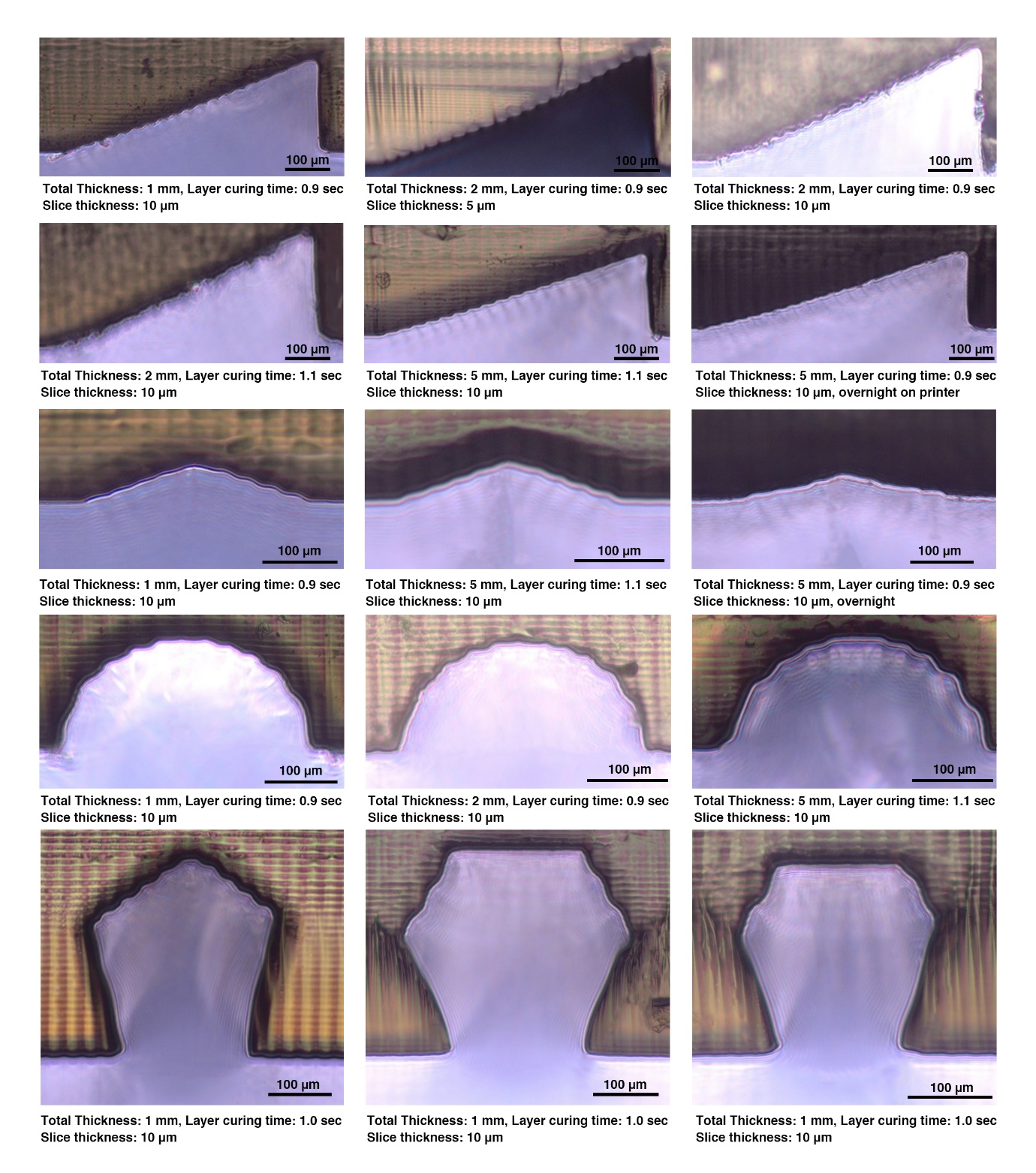


**Fig. S1** The total thickness of a part, curing time of each layer, and slice thickness are the most critical parameters affecting the quality of the channel cross-sections in inertial microfluidic devices. In this study, various cross-sections, ranging from right-angled triangular to pentagonal and hexagonal, were fabricated, and the most optimized parameters were identified. Selecting the optimized parameters lead to fabrication of various cross-sections with high degree of quality and accuracy.


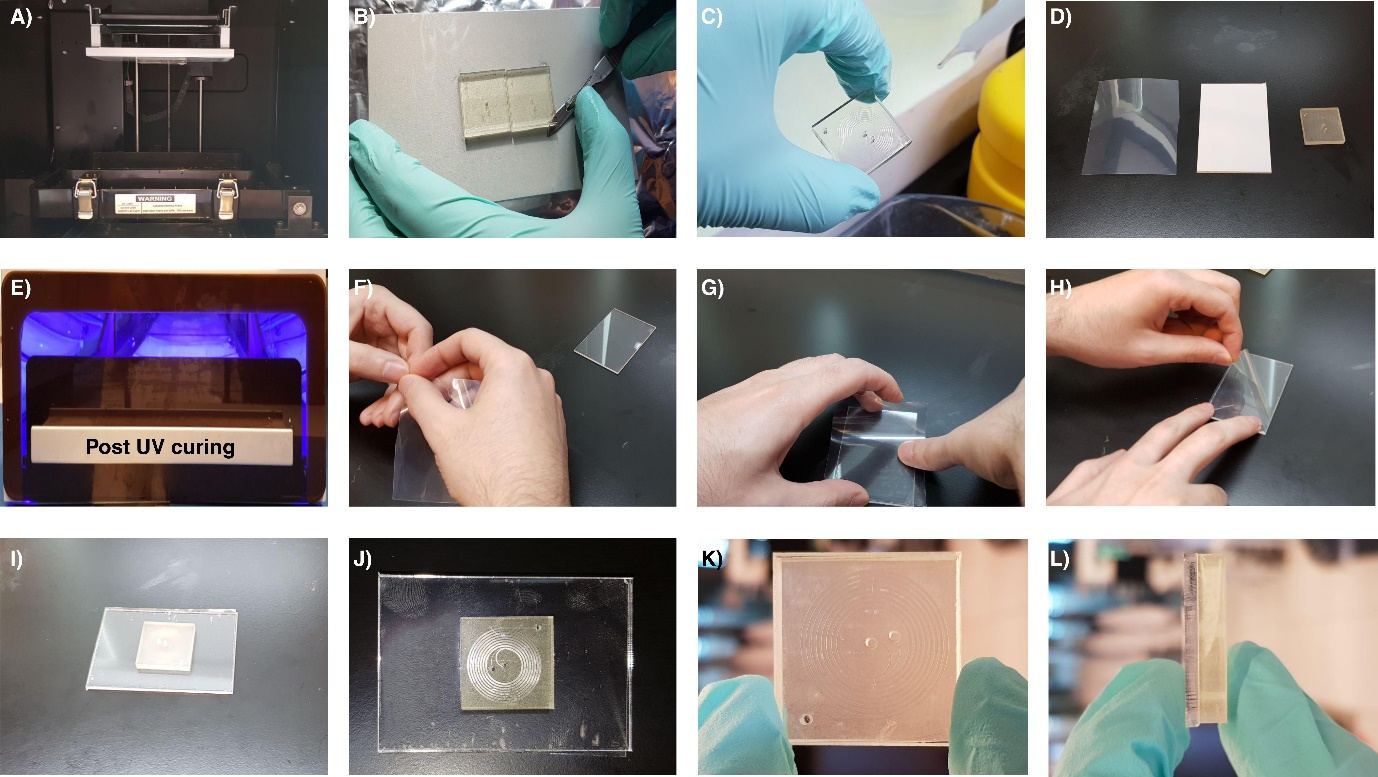


**Fig. S2** The step-by-step fabrication process of an inertial microfluidic device, from printing the part to bonding it to a PMMA sheet.


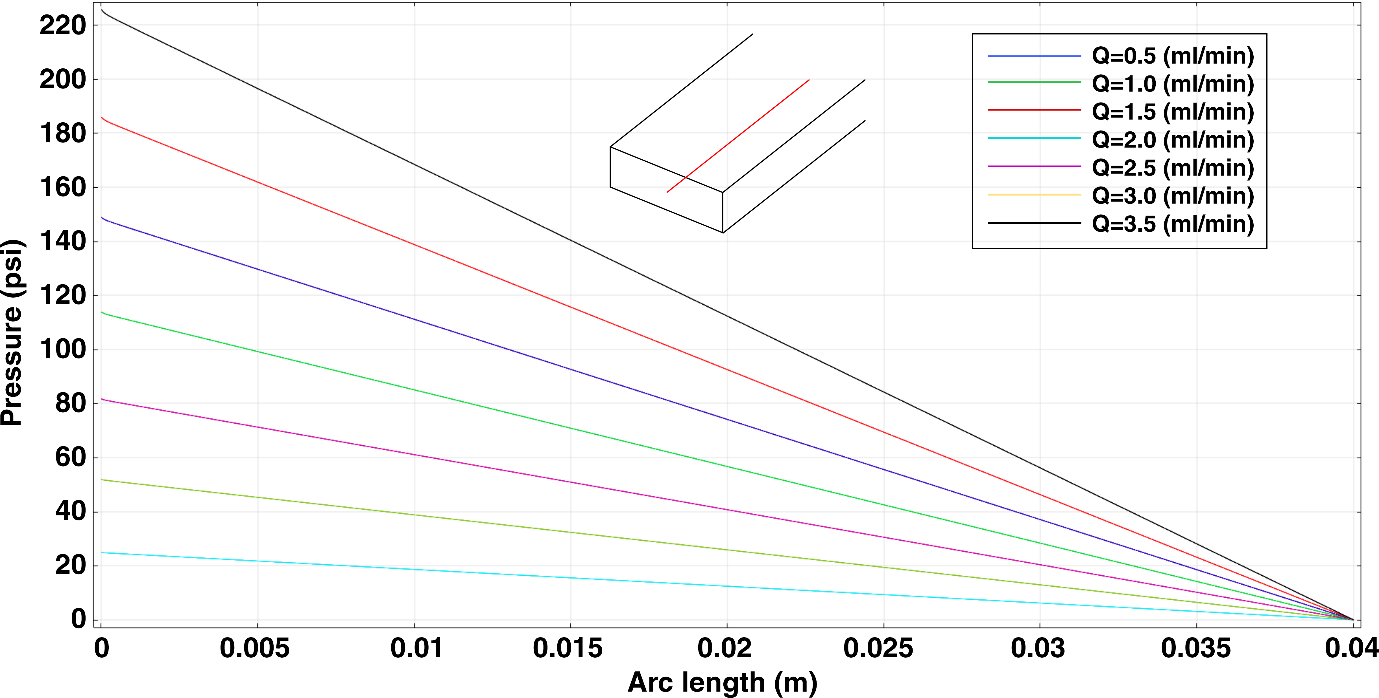


**Fig. S3** The pressure distribution along the length of the channel. Higher flow rates lead to an increase in the amount of applied pressure on the channel. Also, since the channel is straight, the variation of pressure along channel length is linear.


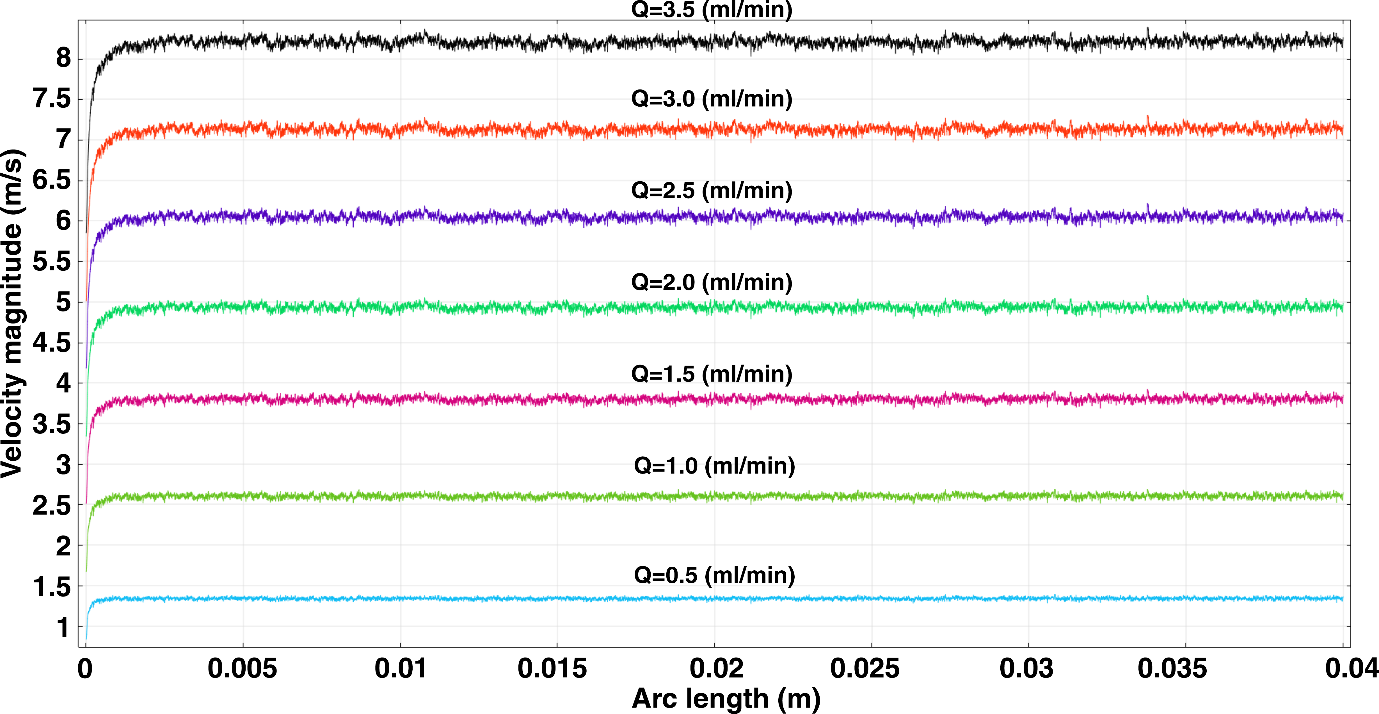


**Fig. S4** velocity distribution along a line passed through the center of the channel.

**Table S1** Specification of rectangular straight microchannel

| **Width** | 200 µm |
| --- | --- |
| **Height** | 50 µm |
| **Aspect ratio** | 4 |
| **Length** | 5 cm |
| **Inlet area** | 0.01 mm^2^ |
| **Inlet perimeter** | 0.5 mm |
| **Hydraulic diameter** | 80 µm |
| **Volume** | 0.47 mm^3^ |

**Table S2** Specification of curvilinear microchannel

| **Main channel width** | 200 µm |
| --- | --- |
| **Height** | 50 µm |
| **Aspect ratio** | 4 |
| **Loops** | 18 |
| **Length** | 41.5 mm |
| **Inlet length** | 30 |
| **Inlet area** | 0.01 mm^2^ |
| **Inlet perimeter** | 0.5 mm |
| **Hydraulic diameter** | 80 µm |
| **Outlet width** | 600 µm |
| **Bifurcation width** | 200 µm |
| **Volume** | 0.82 mm^3^ |

**Table S3** Specification of serpentine microchannel

| **Main channel width** | 200 µm |
| --- | --- |
| **Height** | 40 µm |
| **Aspect ratio** | 5 |
| **Loops** | 15 |
| **Length** | 18.5 mm |
| **Inlet length** | 30 |
| **Inlet area** | 0.008 mm^2^ |
| **Inlet perimeter** | 0.5 mm |
| **Hydraulic diameter** | 64 µm |
| **Volume** | 0.32 mm^3^ |

**Table S4** Specification of contraction-expansion array

| **Main channel width** | 860 µm |
| --- | --- |
| **Height** | 40 µm |
| **Number of CE arrays** | 75 |
| **Length** | 32.9 mm |
| **Inlet length** | 10 mm |
| **Inlet area** | 0.03468 mm^2^ |
| **Inlet perimeter** | 1.814 mm |
| **Hydraulic diameter** | 76.5 µm |
| **Contraction section radius** | 40 µm |
| **Expansion section radius** | 80 µm |
| **Contraction-expansion ratio** | 2 |
| **Volume** | 0.79 mm^3^ |

**Table S5** Specification of spiral microchannel

| **First diameter** | 10.6 mm |
| --- | --- |
| **Last diameter** | 26.6 mm |
| **Turns** | 8 |
| **Width** | 600 µm |
| **Sidewalls** | 80 and 130 µm |
| **Inlet area** | 0.063 mm^2^ |
| **Inlet perimeter** | 1.412 mm |
| **Inner outlet width** | 300 µm |
| **Outer outlet width** | 300 µm |
| **Hydraulic diameter** | 178 µm |
| **Volume** | 29.78 mm^3^ |


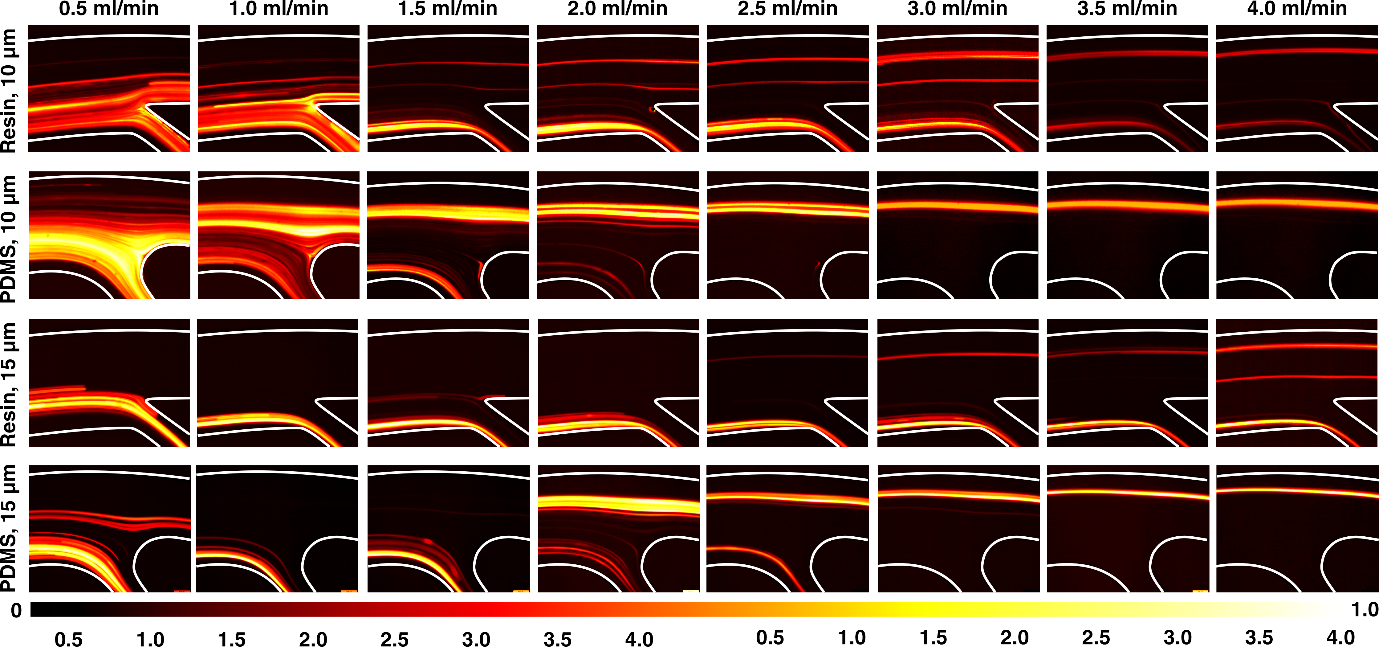


**Fig. S5** Comparison of particle focusing on hard (3D-printed) and soft (PDMS-based) spiral microchannel. The results show that lateral migration of particles in these two devices are not similar, indicating that the softness of PDMS has a significant effect on the focusing behavior of particles

**
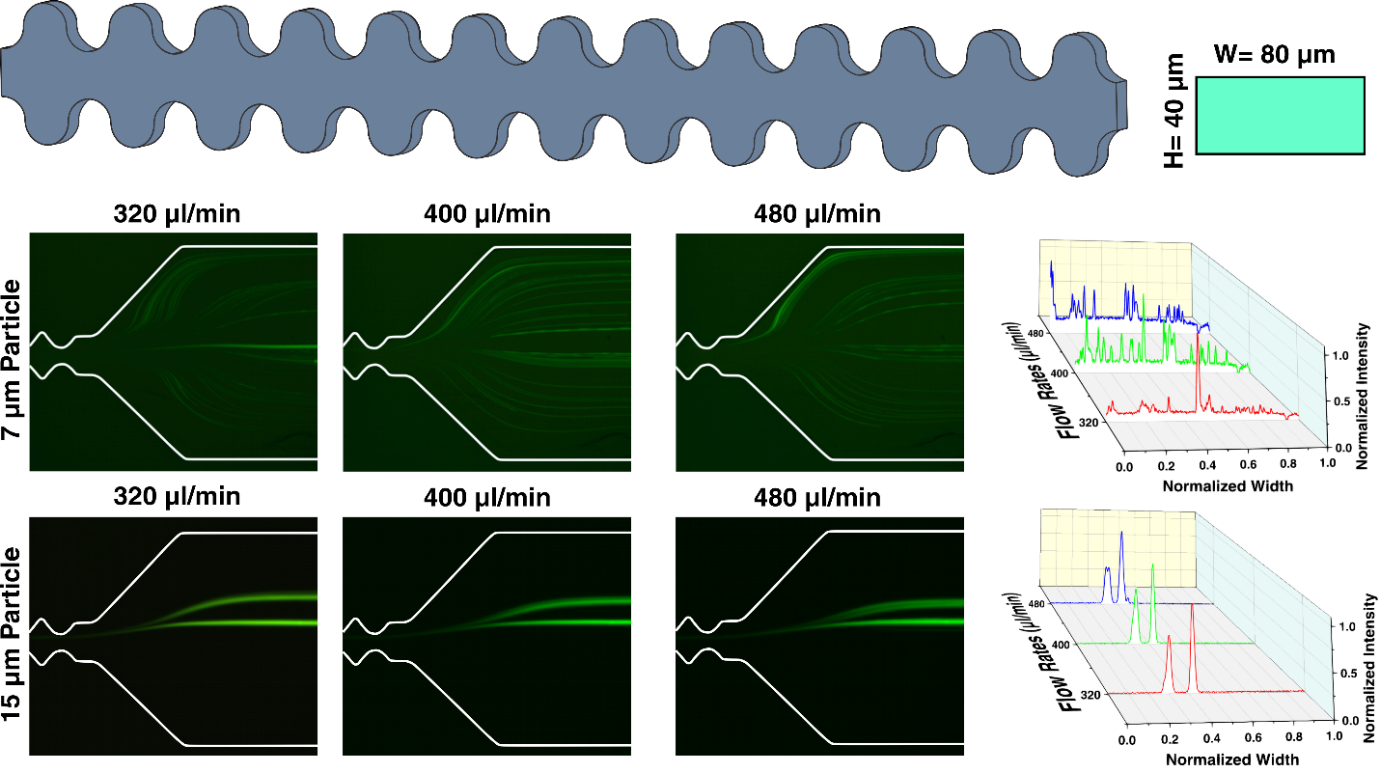
**

**Fig. S6** inertial microfluidics in a contraction-expansion arrays microchannel. Results show that particles with smaller diameter tend to focus on the side of the channel while larger particles usual occupy the center of the channel.

**
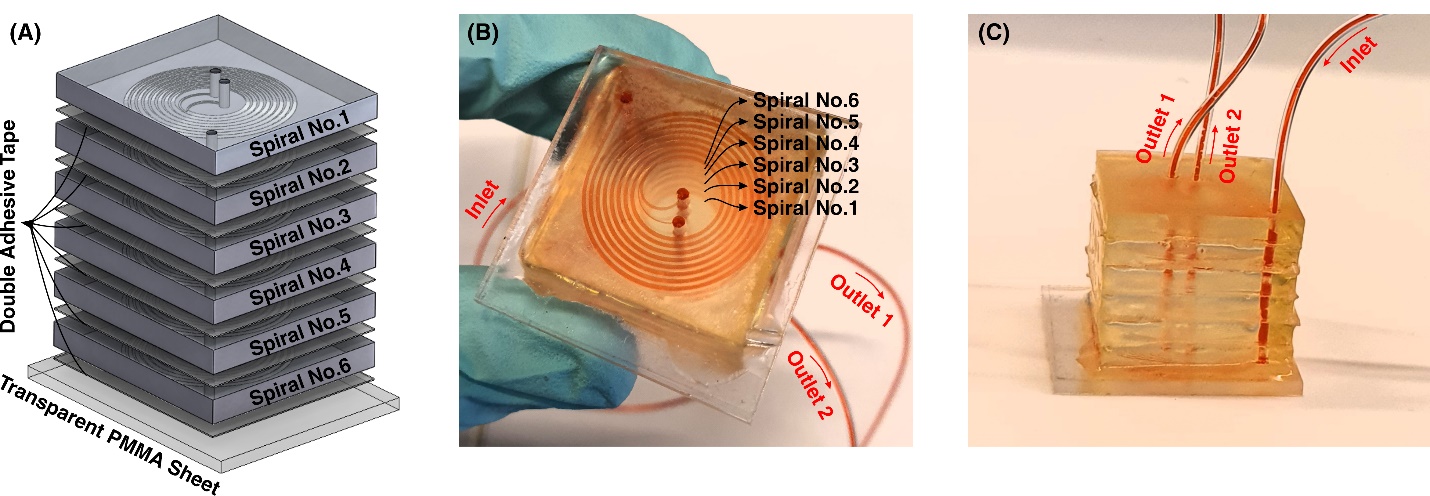
**

**Fig. S7** A) Schematic illustration of a high throughput platform containing multi layers of spiral microchannels. These channels are bonded via a high-pressure double adhesive tape. B) The bottom view and C) the side view of the multiplexed microchannel. Channels are filled with red dyes for better illustration..


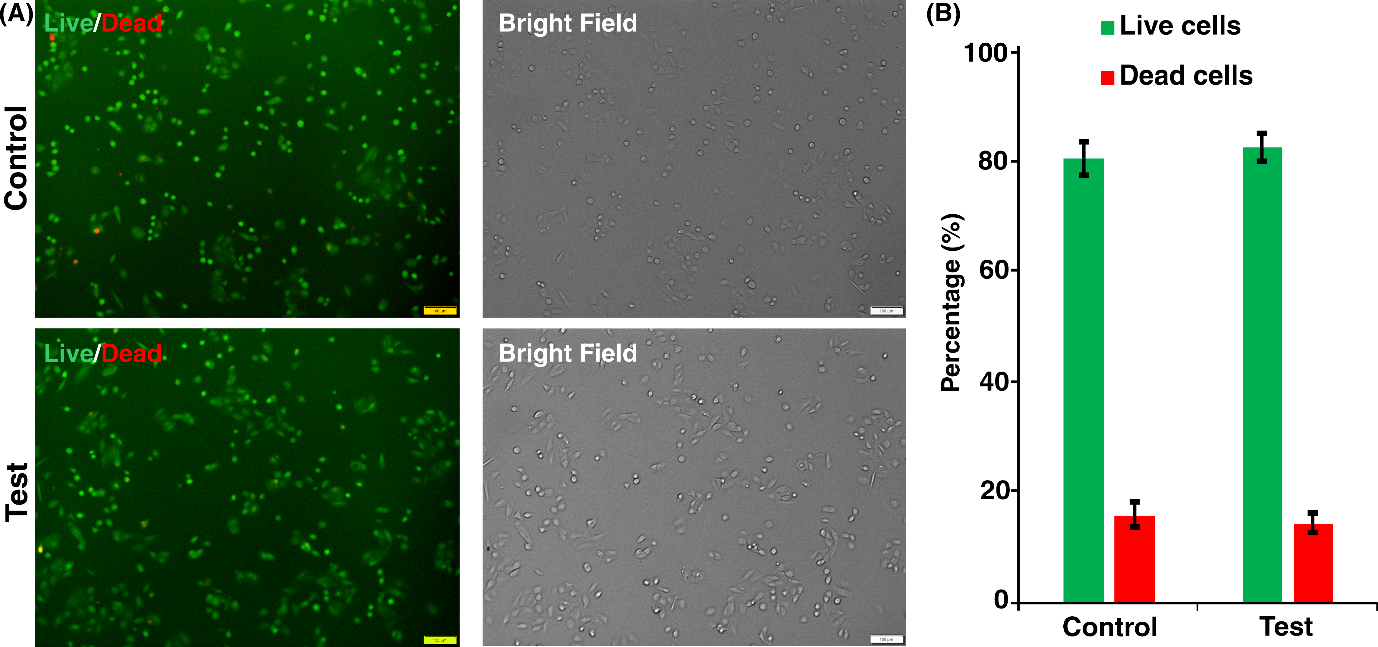


**Fig. S8** A) Microscopic images of cells reveal that there is not any noticeable difference in the live and dead population of cells between the test and control group after one day of experiment. B) The percentage of live and dead cells after passing them through the channel. The results show that there are not any significant differences between the test and control groups.

**References**

1. Razavi Bazaz, S., et al., *Rapid Softlithography Using 3D‐Printed Molds.* Advanced Materials Technologies, 2019: p. 1900425.

2. Wu, Z., et al., *Continuous inertial microparticle and blood cell separation in straight channels with local microstructures.* Lab on a Chip, 2016. **16**(3): p. 532-542.

3. Warkiani, M.E., et al., *Malaria detection using inertial microfluidics.* Lab on a Chip, 2015. **15**(4): p. 1101-1109.

4. Lee, M.G., et al., *Inertial blood plasma separation in a contraction–expansion array microchannel.* Applied Physics Letters, 2011. **98**(25): p. 253702.

5. Bhagat, A.A.S., et al., *Pinched flow coupled shear-modulated inertial microfluidics for high-throughput rare blood cell separation.* Lab on a Chip, 2011. **11**(11): p. 1870-1878.

6. Jiang, D., et al., *Numerical simulation of particle migration in different contraction–expansion ratio microchannels.* Microfluidics and Nanofluidics, 2019. **23**(1): p. 7.

7. Warkiani, M.E., et al., *Membrane-less microfiltration using inertial microfluidics.* Scientific reports, 2015. **5**: p. 11018.

8. Khoo, B.L., et al., *Clinical validation of an ultra high-throughput spiral microfluidics for the detection and enrichment of viable circulating tumor cells.* PloS one, 2014. **9**(7): p. e99409.
